# Prioritizing Service Needs and Willingness to Pay for Aging in Place Among Older Adults in Korea

**DOI:** 10.1093/geroni/igaf122.2836

**Published:** 2025-12-31

**Authors:** Bomgyeol Kim, Hun Kang, Seongmi Choi, JaeWon Hyun, JiYeon Choi

**Affiliations:** Yonsei University College of Nursing, Seoul, Republic of Korea; Yonsei University, Seoul, Republic of Korea; Daegu Catholic University, Daegu, Republic of Korea; Yonsei University, Seoul, Republic of Korea; Yonsei University, Seoul, Republic of Korea

## Abstract

Aging in place (AIP) is key to healthy, independent aging, but sustaining public services is increasingly challenging. Understanding service needs and willingness to pay (WTP) is essential for effective support strategies. Using the 2023 National Survey of Older Koreans, this study classified groups based on service needs and WTP using latent class analysis and examined factors associated with group membership. Service needs and WTP were assessed across six domains: housing support, daily living support, safety support, medical services, transportation support, and counseling services. Among 9,951 community-dwelling older adults, four distinct groups were identified (p<.001): High Needs & High WTP (22.6%), Low Needs & Low WTP (21.0%), Moderate Needs & Moderate WTP (35.1%), and High Needs but Low WTP (21.4%). Individuals with higher education and income were less likely to belong to the low WTP groups. Individuals with poorer self-rated health were more likely to be in the ‘High Needs & High WTP’ group, as were those with three or more chronic diseases or functional limitations. In contrast, individuals with cognitive impairment were more likely to be in the ‘Low Needs & Low WTP’, ‘Moderate Needs & Moderate WTP’, or ‘High Needs but Low WTP’ groups. Individuals who preferred institutional care when experiencing functional dependence were more likely to be classified into the ‘Moderate Needs & Moderate WTP’ and ‘High Needs but Low WTP’ groups. These findings highlight diverse service needs and financial considerations among older adults. Public policies should prioritize affordability and accessibility for low-WTP individuals with high service needs.

